# The Education of the Dairymaid

**Published:** 1897-07-31

**Authors:** 


### MODERN SOCIOLOGY.

### THE EDUCATION OF THE DAIRYMAID.

THE more one travels the more one becomes convinced on coming home that England is the garden of the world. Where else are there such rich pastures, gold-starred with buttercups, enclosed by generous hedges, and dotted with wide-spreading trees? Our towns, busy and numerous as they are, are only black dots, as in the map, sprinkled among the miles of meadow and cornland. And yet our country is one of the largest importers of farm produce in the world. Why is this? Because, for one thing, we are rather thick on the soil, and cannot produce enough for the millions of our land. But if this were the only reason, agriculture should be the most flourishing of all industries, and instead of this we know that acres upon acres are now falling out of cultivation, while over the rest of the country the bitter cry of the farmer ceases not day nor night. We know what the cry is—that the foreigner has invaded the farmyard, and that the native-born cannot make a living as his father did before him. No, certainly not. Let the farmer take it to heart that in this generation no man makes his living as his father did before him. The weaver's son is the manufacturer, and the smith's son is the ironmaster, and every man everywhere has called in science to aid manual skill. So must the farmer do if he also is to make a living.

It is not wholly the farmer's fault if he does not do so, for he gets but little education in the chemistry of his trade. In technical education all round we are not alongside our neighbours—not to say ahead—and in agricultural education we are very far behind. As one observer* remarks: "The little kingdom of Wurtemberg, which is about the size of a large British county, spends more on its own account (and quite independently of the imperial expenditure) on agricultural education than the Board of Agriculture of the wealthiest country in the world." What this nation—this fertile Britain—spends on teaching its children to increase its fertility is £8,000 per annum. The sum is not excessive. It would be difficult to name any object of national interest on which less is spent. Perhaps agriculture is not a subject of national interest. So much the worse, for it certainly ought to be. Man cannot live without bread. At present we can import wheat, pork, eggs, and butter either cheaper or better than we can produce it ourselves; in some cases both cheaper and better. But suppose a great war in which our ports were blockaded—may heaven defend us from such misfortune, and "weel may the keel row" of the ironclads that averts it—what would happen to our imports then? The farmer might rejoice, but how many millions would have to stint, how many thousands would starve, while his pockets jingled with unfamiliar gold: for, in the ultimate result, the farmer is the monarch of us all, and our emergency is his opportunity. Until the emergency comes, he asks for the artificial war of protection. We wish the farmer well, but we do not want to enrich him by impoverishing the rest of the nation. Protection! What other protection does he need than proximity to his markets? The foreigner always lies under the disadvantage of distance. Yet we import wheat from America, Argentina, Hungary, and Russia; fowls and eggs from France; beef from Canada; mutton from New Zealand; fruit from Holland; butter, and even milk, frozen into solid blocks, from Denmark. Why should these things be?

* Mr. James Hendrick, B.Sc., Glasgow Technical College.

The common explanation is that in England wages are so high. Yet the agricultural labourer is probably the worst paid of all British workmen. This hypothesis, even if well founded, will not explain all the phenomena. At the root of the matter lies the fact that the British farmer does not know his business as well as the foreigner, and does not get the chance to learn it. The British Government indeed spends £8,000 in teaching the principles of agriculture in various colleges. But France spends as much on the support of the Institut National Agronomique in Paris alone; Germany spends twice as much on the Agricultural High School at Berlin—twice as much on a single school as Britain spends on all it has! In the little town of Guelph in Ontaria there is an agricultural college which might give a lesson to almost any in the old country, and all American farming is conducted on principles of concentration and labour-saving, which take away something from the picturesqueness of farming, but add immensely to its profitableness.

The British farmer may be clever at his work in an empirical way—the way that breeds conceit and unwillingness to change—but he knows little of the science of his craft. How can he compete with a Teuton who has been trained at Leipzig? In Leipzig there are nine professors, who give lectures on strictly agricultural subjects, *i.e.*, practical agriculture and dairying, agricultural chemistry, agricultural botany, forestry, veterinary science, agricultural mechanics and engineering, agricultural bacteriology, and agricultural book-keeping. In addition to these there are 24 lecturers dealing with subjects less directly connected with farming, such as geology, mineralogy, physiology, agricultural geography, the history of the land laws, the history of peasants, national economy and finance, colonial expansion, and communism. There are chemical, physiological, and botanical laboratories, a field for experiments, a dairy, and a veterinary hospital. At Halle, 25 miles from Leipzig, is an even larger school, with an agricultural geological garden attached to it.

We are at last realising our error and setting up agricultural colleges, but it will take a long time to recover the position that has been lost. Since dairy instruction has been introduced into Ireland, Irish butter is beginning to recover the position it has lost. But it is often sold as Danish butter, because Irish butter has got a bad name! Still, to regain a lost market, even under a false name, is better than to lose it for ever. Money spent in dairy instruction seems to be well spent. The County Council of Westmoreland reckoned that if, thanks to improved instruction in the art, they could improve the quality of their butter 1d. per lb., the gain to the country would be 5,000 per cent. on what it is spending on teaching butter-making. Such a gain is by no means uncommon, and improvements of 2d. and 3d. per lb. have been reported as the result of dairy education. With cheese, an improvement in price of 10s. per cwt. is recorded as having resulted from better teaching. It has been estimated that if the prices of agricultural products could be raised only one per cent. the result would be a gain of three million pounds annually to the country's wealth. It is the farmer's business, it will be his profit, to make it so.

